# HOTAIR regulates osteogenic–adipogenic differentiation imbalance through the miR-217/DKK1-mediated Wnt/β-catenin pathway in SONFH-BMSCs

**DOI:** 10.3389/fgene.2026.1917162

**Published:** 2026-09-29

**Authors:** Min Yi, Feifei Lin, Zhenwu Du, Qingyu Wang

**Affiliations:** 1 Department of Orthopedics, The Second Hospital of Jilin University, Jilin University, Changchun, Jilin, China; 2 Joint International Research Laboratory of Aging Active Strategy and Bionic Health in Northeast Asia of Ministry of Education, Jilin University, Changchun, Jilin, China

**Keywords:** DKK1, HOTAIR, mesenchymal stem cells, miR-217, osteogenesis, steroid-induced osteonecrosis of the femoral head, Wnt/β-catenin signaling

## Abstract

**Background:**

Steroid-induced osteonecrosis of the femoral head (SONFH) is characterized by disrupted osteogenic and adipogenic differentiation of bone marrow mesenchymal stem cells (BMSCs). The long noncoding RNA HOTAIR is aberrantly upregulated in SONFH-BMSCs, yet its functional role in the differentiation imbalance remains incompletely understood. In this study, we investigated whether HOTAIR silencing could restore the differentiation balance in SONFH-BMSCs and clarify the underlying regulatory mechanism.

**Methods:**

HOTAIR was knocked down in SONFH-BMSCs using small interfering RNA (si-HOTAIR). Osteogenic differentiation was assessed using alkaline phosphatase (ALP) and Alizarin Red S (ARS) staining, while adipogenic differentiation was evaluated using Oil Red O staining. Expression of lineage-specific markers and Wnt pathway components was measured using qRT-PCR and Western blotting. The interactions among HOTAIR, miR-217, and DKK1 were validated through dual-luciferase reporter and rescue experiments.

**Results:**

HOTAIR silencing significantly enhanced osteogenic differentiation, as evidenced by increased ALP activity, mineralization, and upregulation of RUNX2, BMP2, and Osterix, while concurrently suppressing adipogenic differentiation, with reduced lipid droplet formation and downregulation of PPARγ, C/EBPα, and Adipsin. Mechanistically, HOTAIR acted as a candidate competing endogenous RNA (ceRNA) by sponging miR-217, thereby relieving miR-217-mediated repression of DKK1 and inhibiting Wnt/β-catenin signaling. HOTAIR knockdown restored miR-217 availability, decreased DKK1 expression, and enhanced β-catenin expression.

**Conclusion:**

HOTAIR inhibition rebalances osteogenic and adipogenic differentiation of SONFH-BMSCs via the miR-217/DKK1/Wnt/β-catenin axis. These findings identify HOTAIR as an important molecular regulator of BMSC fate determination and provide mechanistic insights into ceRNA-mediated regulation in SONFH pathogenesis.

## Background

Steroid-induced osteonecrosis of the femoral head (SONFH), characterized by inadequate blood flow and subsequent femoral head death, is caused by prolonged or high-dose administration of hormonal medications, such as glucocorticoids (Chang et al., 2020). Patients typically exhibit clinical manifestations, including hip discomfort, restricted mobility, and irregularities in gait (Zhao et al., 2020). SONFH is characterized by a high disability rate and rapid course of disease. Conservative treatment is generally ineffective and can enhance patients’ outlook and extend the lifespan of joints. However, available therapeutic options are currently restricted because of an incomplete understanding of the underlying causes (Huang et al., 2021).

Research has indicated that SONFH affects the quantity and performance of BMSCs (Houdek et al., 2016). SONFH is primarily caused by decreased osteogenic activity and increased adipogenic differentiation of BMSCs, which are considered core pathogenic factors of the disease (Wang et al., 2019). BMSCs possess robust proliferative capacity, multidirectional differentiation potential, immunomodulatory properties, and genetic modifiability. They have demonstrated significant therapeutic potential in various diseases, including musculoskeletal, cardiovascular, nervous system, and autoimmune disorders (Murphy et al., 2013). In particular, the osteogenic differentiation capacity of BMSCs has been widely applied in the early treatment of SONFH (Huang et al., 2023; Shu et al., 2020).

The extensive use of next-generation sequencing technologies in transcriptomics has demonstrated the significant role of long noncoding RNAs (lncRNAs) in regulating the osteogenic differentiation of BMSCs (Yang et al., 2018). LncRNAs are non-protein-coding RNA molecules longer than 200 nucleotides that act as regulatory molecules to control multiple physiological processes, including cell proliferation, differentiation, and apoptosis (Mattick et al., 2023). Advancements in detection technology and epigenetics have revealed an increasing number of lncRNAs that are linked to several human disorders, including cancers (Li et al., 2025), along with cardiovascular (Boon et al., 2016), degenerative (Tuerlings et al., 2022), and metabolic diseases (Brandt and Kopp, 2024). Multiple studies have demonstrated that lncRNA dysregulation can result in the activation or inhibition of cellular pathways that control BMSC development.

HOTAIR, a well-characterized lncRNA located on chromosome 12q13.13, contains seven exonic regions. LncRNA-HOTAIR is recognized as the first identified trans-acting lncRNA due to its ability to repress HOXC gene expression (Rinn et al., 2007). HOTAIR plays a regulatory role in various diseases, including neoplastic diseases, such as lung, cervical, and esophageal cancers (Li et al., 2017; Cáceres-Durán et al., 2020; Abdeahad et al., 2019), as well as in non-neoplastic diseases, such as neurodegenerative diseases and osteoarthritis (Ahmad et al., 2024; Ajekigbe et al., 2019). Additionally, HOTAIR is involved in regulating osteogenic differentiation of BMSCs. Studies have shown that HOTAIR impedes BMSC osteogenic differentiation by modulating the Wnt/β-catenin signaling pathway, ultimately contributing to osteoporosis development (Shen et al., 2019). Furthermore, it regulates bone formation and proliferation of BMSCs from patients with non-traumatic femoral head necrosis by sponging miR-17-5p and targeting its downstream gene SMAD7 (Wei et al., 2017).

We previously reported markedly elevated HOTAIR expression in BMSCs from patients with SONFH (Wang et al., 2018). Based on this observation, we aimed to further elucidate the functional role and molecular mechanism of HOTAIR in the disrupted osteogenic/adipogenic differentiation balance that characterizes SONFH pathogenesis. In particular, we sought to determine whether HOTAIR silencing could restore osteogenic commitment and suppress excessive adipogenesis in patient-derived BMSCs and to identify the downstream ceRNA regulatory network involved. This study therefore identifies HOTAIR as a functionally relevant regulator of BMSC differentiation imbalance in SONFH and provides mechanistic insights into the underlying ceRNA network.

## Methods

### Study design and participants

Eleven patients with SONFH (6 male and 5 female patients, 67.09 ± 4.99 years of age) and 10 patients with femoral neck fracture (FNF) (4 male and 6 female females, 71 ± 7.94 years of age) were enrolled from the Department of Orthopedics, the Second Hospital of Jilin University, China. The diagnosis of SONFH was confirmed using X-ray, CT, and MRI, and all SONFH patients had a confirmed history of glucocorticoid exposure (continuous oral prednisone ≥20 mg/day for ≥21 days, cumulative prednisone-equivalent dose ≥1,000 mg via intermittent high-dose therapy, or cumulative dose >2000 mg within 3 months). Exclusion criteria included non-glucocorticoid-induced femoral head necrosis, concomitant hip disorders, malignancies, autoimmune diseases, severe hepatic/renal dysfunction, poorly controlled diabetes, previous hip surgery, and severe trauma/infection/psychiatric disorders. This study was approved by the Ethics Committee of the Second Hospital of Jilin University (Approval No. 2020/NO.2020080), and written informed consent was obtained from all participants.

### Isolating and culturing BMSCs

BMSCs were isolated from the bone marrow of proximal femurs of patients by density gradient centrifugation (Colter et al., 2000). In brief, samples were diluted with sterile phosphate-buffered saline (PBS) and centrifuged at 1,200 rpm for 10 min at 4 °C. After removal of the supernatant and lipid layer, the cell suspension was centrifuged again at the same speed for 5 min. Mononuclear cells were collected from the buffy coat layer and seeded in 10-cm culture dishes. Cells were cultured in Dulbecco’s modified Eagle’s medium (DMEM, Gibco, NY, United States) supplemented with 10% fetal bovine serum (FBS, Gibco) and 1% penicillin‒streptomycin solution (Pen/Strep, Gibco) at 37 °C in a humidified incubator with 5% CO_2_. The medium was replaced every 3 days. Cells at passages 3–5 were used for subsequent experiments. BMSC identity was confirmed by flow cytometry (surface markers: CD73^+^, CD90^+^, CD105^+^, CD34^−^, and CD45^−^) and multipotent differentiation potential (osteogenic/adipogenic induction assays).

### Plasmids and transfection

siRNAs targeting human HOTAIR (si-HOTAIR), negative control siRNA (si-NC), HOTAIR overexpression vector (ov-HOTAIR) and its control (ov-control), miR-217 mimics, miR-217 inhibitor, and their respective negative controls were synthesized by GenePharma (Shanghai, China). Transfection was performed using Golden Tran-DR reagent (Golden Transfer Technology, Changchun, China). BMSCs were seeded on 6-well plates at a density of 1.5–2.0 × 10^5^ cells per well and cultured until reaching approximately 60%–70% confluence. For siRNA and miRNA transfection, cells were transfected with 60 pmol siRNA, miRNA mimics, or miRNA inhibitors using 4 μL transfection reagent per well. For plasmid transfection, 2 μg plasmid DNA was mixed with 4 μL transfection reagent per well. Transfection efficiency was evaluated using qRT-PCR at 24–48 h post-transfection. Sequences are listed in Table 1.

**TABLE 1:** Sequences of siRNA and miRNA.

Molecule	Sequence (5′→3′)
si-HOTAIR	UUA​AGU​CUA​GGA​AUC​AGC​ACG​AAG​C
si-NC	CGG​AUC​AGC​UCG​CGC​UAU​CAU​CGC​A
miR-217 mimic	UAC​UGC​AUC​AGG​AAC​UGA​UUG​GA
NC-mimic	CGU​GAC​UGU​CAG​AGU​UAG​AGA​GC
anti-miR-217	UCC​AAU​CAG​UUC​CUG​AUG​CAG​UA
anti-NC	CUU​CGU​GCA​GCC​UGA​GUG​UGC​UG

### Luciferase reporter assay

Wild-type (WT) and mutant-type (MUT) sequences of HOTAIR and the DKK1 3′-UTR containing the predicted miR-217 binding sites were cloned into luciferase reporter vectors. HEK-293T cells were co-transfected with WT or MUT plasmids, along with miR-217 mimics or mimic NC. After 24 h, luciferase activity was measured using the Dual-Luciferase Reporter Assay System (Promega, Madison, WI, United States). The relative luciferase activity was normalized to Renilla luciferase activity.

### Osteogenic and adipogenic differentiation

BMSCs at passages 3–5 were seeded at a density of 5 × 10^4^ cells/cm^2^ and cultured until 80%–90% confluence. Osteogenic differentiation was induced by culturing BMSCs in osteogenic medium containing 10% FBS, 10 mmol/L β-glycerophosphate, 0.1 μmol/L dexamethasone, and 50 μg/mL ascorbate (Cyagen Biosciences, Guangzhou, China). To induce adipogenic differentiation, BMSCs were cultured in adipogenic medium containing 10% FBS, 2 μg/mL insulin, 1 μmol/L dexamethasone, 1 μmol/L rosiglitazone, 100 μg/mL 3-isobutyl-1-methylxanthine, and 10 mmol/L glutamine (Cyagen Biosciences). Differentiation media were prepared according to the manufacturer’s instructions and replaced every 2–3 days throughout the induction period. Osteogenic differentiation was evaluated at days 3 and 7 using alkaline phosphatase (ALP) staining and at days 7 and 14 using Alizarin Red S (ARS) staining. Adipogenic differentiation was evaluated at days 7 and 14 using Oil Red O staining. All osteogenic induction reagents were stored in single-use aliquots at −20 °C, and β-glycerophosphate was freshly added to the complete medium before each medium change.

### ALP, ARS, and Oil Red O staining and their quantification

For ALP staining, BMSCs were fixed with 4% paraformaldehyde for 10 min and stained using a BCIP/NBT kit (Beyotime) following the manufacturer’s instructions. ALP activity was measured using p-nitrophenyl phosphate (pNPP; Beyotime), normalized to total protein content (BCA assay; Beyotime), and expressed as nmol/(min·mg). For ARS staining, cells were fixed in 4% paraformaldehyde at 4 °C for 15 min and stained with ARS solution for 15 min; after extensive washing with distilled water, the calcium-bound Alizarin Red S was extracted using 10% cetylpyridinium chloride (CPC) solution for 30 min at room temperature. The absorbance of the extracted solution was measured at 562 nm using a microplate reader to quantitatively evaluate mineralization. For Oil Red O staining, cells were fixed in 4% neutral-buffered formalin for 30 min and stained with Oil Red O (Beyotime) for 1 h; lipid accumulation was quantified by measuring optical density at 490 nm after isopropanol elution. All stained samples were examined under a light microscope (Leica, United States).

### Western blot (WB) analysis

Total cellular protein was extracted with RIPA lysis buffer (Beyotime, China) and quantified using a BCA assay kit (Beyotime, China). Equal protein amounts were separated using SDS-PAGE and transferred to polyvinylidene difluoride (PVDF) membranes (Millipore, United States). The membranes were blocked with 5% non-fat milk in Tris-buffered saline with 0.1% Tween-20 (TBST) for 1 h at room temperature and then incubated overnight at 4 °C with primary antibodies targeting Osterix (Boster, Cat. No. M02077, 1:1,000), BMP2 (Boster, Cat. No. BM4960, 1:1,000), RUNX2 (Boster, Cat. No. BM4700, 1:1,000), PPARγ (Boster, Cat. No. M00449-2, 1:1,000), C/EBPα (Boster, Cat. No. A00386-1, 1:1,000), Adipsin (Boster, Cat. No. M01134, 1:1,000), DKK1 (Boster, Cat. No. A09360-1, 1:1,000), OCT4 (Abmart, Cat. No. T55781, 1:10,000), β-catenin (Abmart, Cat. No. M24002, 1:10,000), and β-actin (Boster, Cat. No. BM3873, 1:10,000). After washing with TBST, the membranes were incubated with HRP-conjugated goat anti-rabbit IgG (Huabio, Cat. No. HA1001, 1:80,000) or goat anti-mouse IgG (Huabio, Cat. No. BA1055, 1:50,000) for 1 h at room temperature. Protein bands were visualized using an enhanced chemiluminescence system (Azure c500; Azure Biosystems, United States). All experiments were performed with at least three independent biological replicates.

### qRT-PCR

Total RNA, including small RNAs, was extracted from BMSCs using TRIzol reagent (Invitrogen, United States). Reverse transcription was performed using the PrimeScript RT Reagent Kit with gDNA Eraser (TaKaRa, Japan) for mRNA and a miRNA-specific reverse transcription kit (Ribo Biosciences, China) with stem-loop primers for miR-217, following the manufacturers’ protocols. qRT-PCR was carried out using FastStart Universal SYBR Green Master Mix (Roche, Switzerland) on an Applied Biosystems 7500 Fast Real-Time PCR System (United States). Data were normalized to GAPDH (for mRNA) or U6 (for miR-217), and relative expression levels were calculated using the 2^-△△Ct^ method. All experiments were performed in triplicate. The primer sequences are listed in Table 2.

**TABLE 2:** Primers for qRT-PCR analyses.

Gene	Primer sequence
HOTAIR	F: 5′- AAA​AAG​CTT​GGG​TTA​TAG​GAA​AGC​C-3′
	R: 5′- GCT​TCT​ATG​TTC​CTC​TCA​AAT​TCC​G-3′
BMP2	F: 5′- GGA​ATG​ACT​GGA​TTG​TGG​CT-3′
	R: 5′- TGA​GTT​CTG​TCG​GGA​CAC​AG-3′
Osterix	F: 5′- TGC​TTG​AGG​AGG​AAG​TTC​AC-3′
	R: 5′- AGG​TCA​CTG​CCC​ACA​GAG​TA -3′
RUNX2	F: 5′- AGA​TGG​GAC​TGT​GGT​TAC​TG-3′
	R: 5′- GTA​GCT​ACT​TGG​GGA​GGA​TT-3′
Adipsin	F: 5′- TGC​TAC​AGC​TGT​CGG​AGA​AG -3′
	R: 5′- ATG​ACT​TCA​TTG​CTC​GGG​AC -3′
C/EBPα	F: 5′- GGGCAAGGCCAAGAAGTC -3′
	R: 5′- TTG​TCA​CTG​GTC​AGC​TCC​AG -3′
PPARγ	F: 5′- GAG​CCC​AAG​TTT​GAG​TTT​GC -3′
	R: 5′- CTG​TGA​GGA​CTC​AGG​GTG​GT-3′
DKK1	F: 5′- ACA​CTT​CTG​GTC​CAA​GAT​CTG​TAA​A-3′
	R: 5′- ACG​CTG​GAA​TAT​TTC​TAG​TCC​ATG​A-3′
miR-217	F: 5′-CGC​TCT​ACT​GCA​TCA​GGA​ACT​GA-3′
	R: 5′-GTG​CAG​GGT​CCG​AGG​T-3′
β-catenin	F: 5′- TGC​TGA​AGG​TGC​TGT​CTG​TC -3′
	R: 5′- CTG​CTT​AGT​CGC​TGC​ATC​TG -3′
Oct4	F: 5′- CCG​GCA​TTT​GCT​ATA​ACC​GC -3′
	R: 5′- CCT​GCC​ATC​CCG​AAT​ATC​CC -3′
U6	F: 5′-GCT​TCG​GCA​GCA​CAT​ATA​CTA​AAA​T-3′
	R: 5′-CGC​TTC​ACG​AAT​TTG​CGT​GTC​AT-3′
Actin	F: 5′- TGA​CGT​GGA​CAT​CCG​CAA​AG-3′
	R: 5′- CTG​GAA​GGT​GGA​CAG​CGA​GG-3′

### Statistical analysis

For all cell-based functional and mechanistic assays, data were obtained from at least three independent biological replicates. Where applicable, these replicates were derived from independent donors, and each biological replicate included three technical replicates. For dual-luciferase reporter assays, three independent transfections were performed. Data are presented as mean ± standard deviation (SD). Two-group comparisons were performed using a two-tailed Student's t-test, whereas one-way analysis of variance (ANOVA) followed by Tukey’s *post hoc* test was used for multiple-group comparisons. All statistical analyses were performed using GraphPad Prism 9. Significance levels were set at *P < 0.05, **P < 0.01, and ***P < 0.001.

## Results

### Baseline characteristics of the study population

A total of 11 patients with SONFH (Steinberg stages IV–V) and 10 patients with FNF serving as controls were enrolled in this study. The demographic and clinical characteristics of the study population are summarized in Table 3. No statistically significant differences were observed between the SONFH and control groups with respect to age, body mass index (BMI), inflammatory markers [C-reactive protein (CRP) and erythrocyte sedimentation rate (ESR)], or sex distribution (all P > 0.05). These findings suggest that the two groups were comparable at baseline, thereby reducing the likelihood that observed experimental differences were attributable to demographic or systemic inflammatory confounders.

**TABLE 3:** Characteristics of the patients in this study. BMI, body mass index; CRP, C-reactive protein; ESR, erythrocyte sedimentation rate.

Variable	SONFH (n = 11)	Control (n = 10)	P-value
Age (years)	67.09 ± 4.99	71 ± 7.94	0.188
Gender (M/F)	6/5	4/6	-
BMI (kg/m^2^)	24.2 ± 2.93	25.87 ± 3.45	0.25
CRP (mg/dL)	3.23 ± 1.99	1.77 ± 2.99	0.21
ESR (mm/h)	26 ± 20.47	26.82 ± 21.89	0.93
Steinberg staging system (IV/V)	3/8	-	-
Femoral head collapse (mm)	9.76 ± 7.26	-	-
Harris score	55 ± 5	-	-

### Characterization of BMSCs and aberrant HOTAIR upregulation in SONFH patient-derived BMSCs

BMSCs from both groups were successfully isolated and stably expanded *in vitro*. Under phase-contrast microscopy, cells in both groups displayed the typical spindle-shaped, fibroblast-like morphology characteristic of mesenchymal stem cells, growing as adherent monolayers with uniform morphology across successive passages (Figure 1A). Flow cytometric analysis confirmed that all isolated cells were negative for the hematopoietic markers CD34 and CD45 (Figure 1B) and constitutively expressed the canonical mesenchymal surface antigens CD73, CD90, and CD105 (Figure 1C). These immunophenotypic features are fully consistent with the defining criteria for mesenchymal stem cells established by the International Society for Cellular Therapy (ISCT).

**FIGURE 1:**
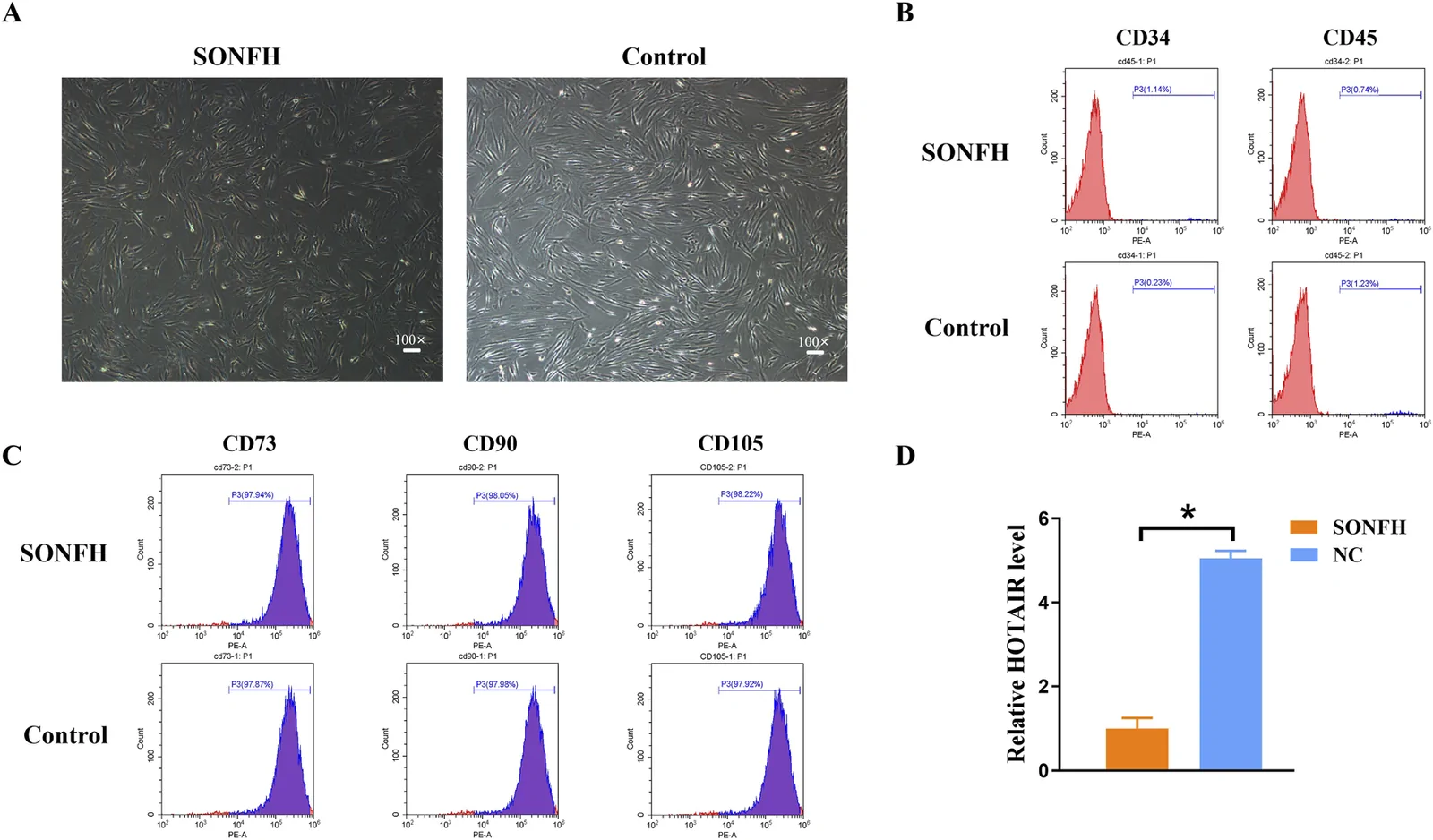
Morphological and phenotypic characterization of BMSCs from SONFH patients and controls. **(A)** Morphological observation of BMSCs under light microscopy. **(B)** Flow cytometry analysis showing the absence of hematopoietic surface markers (CD34 and CD45) in BMSCs from both groups. **(C)** Flow cytometry analysis confirming the expression of mesenchymal stem cell surface markers (CD73, CD90, and CD105) in BMSCs from both groups. **(D)** Relative HOTAIR mRNA levels in BMSCs from SONFH patients (n = 11) and NC (n = 10), detected using qRT-PCR with GAPDH as the internal reference. HOTAIR expression is significantly elevated in SONFH-BMSCs. Data are presented as mean ± SD. Statistical significance was assessed using a two-tailed Student's t-test. **P < 0.01.

Given our previous microarray finding that HOTAIR is among the most dysregulated lncRNAs in SONFH-BMSCs (Wang et al., 2018), we verified its expression level in the entire study cohort (n = 11 SONFH, n = 10 controls) using qRT-PCR. As presented in Figure 1D, HOTAIR transcript levels were significantly higher in BMSCs from SONFH patients than in those from controls (P < 0.01). This independent validation corroborates the aberrant upregulation of HOTAIR in SONFH-BMSCs, implicating it in the pathological differentiation imbalance and providing a rationale for the subsequent loss-of-function mechanistic investigations.

### HOTAIR silencing promotes osteogenic differentiation and inhibits adipogenic differentiation in SONFH-BMSCs

To evaluate whether HOTAIR contributes to the impaired differentiation phenotype of SONFH-BMSCs, osteogenic and adipogenic differentiation assays were performed using four groups: FNF-derived control BMSCs (NC), untreated SONFH-BMSCs, SONFH-BMSCs transfected with si-NC, and SONFH-BMSCs transfected with si-HOTAIR. All experiments were performed using BMSCs from three independent biological replicates. qRT-PCR confirmed that si-HOTAIR significantly reduced HOTAIR expression in SONFH-BMSCs (P < 0.01, Supplementary Figure S1).

Compared with FNF-derived control BMSCs, SONFH-BMSCs exhibited reduced osteogenic differentiation capacity. HOTAIR silencing significantly enhanced osteogenic differentiation compared with untreated SONFH-BMSCs and si-NC controls. (Figures 2A,B). ARS staining and quantification on days 7 and 14 demonstrated that si-HOTAIR significantly promoted mineralization compared with untreated SONFH-BMSCs and si-NC controls (P < 0.001; Figures 2C,D). qRT-PCR analysis showed that si-HOTAIR significantly upregulated the mRNA expression of the osteogenic markers Osterix, BMP2, and RUNX2 compared with untreated SONFH-BMSCs and si-NC controls (all P < 0.001; Figure 2E). Western blot analysis confirmed that the protein levels of Osterix, BMP2, and RUNX2 were consistent with the mRNA expression patterns (Figure 2F).

**FIGURE 2:**
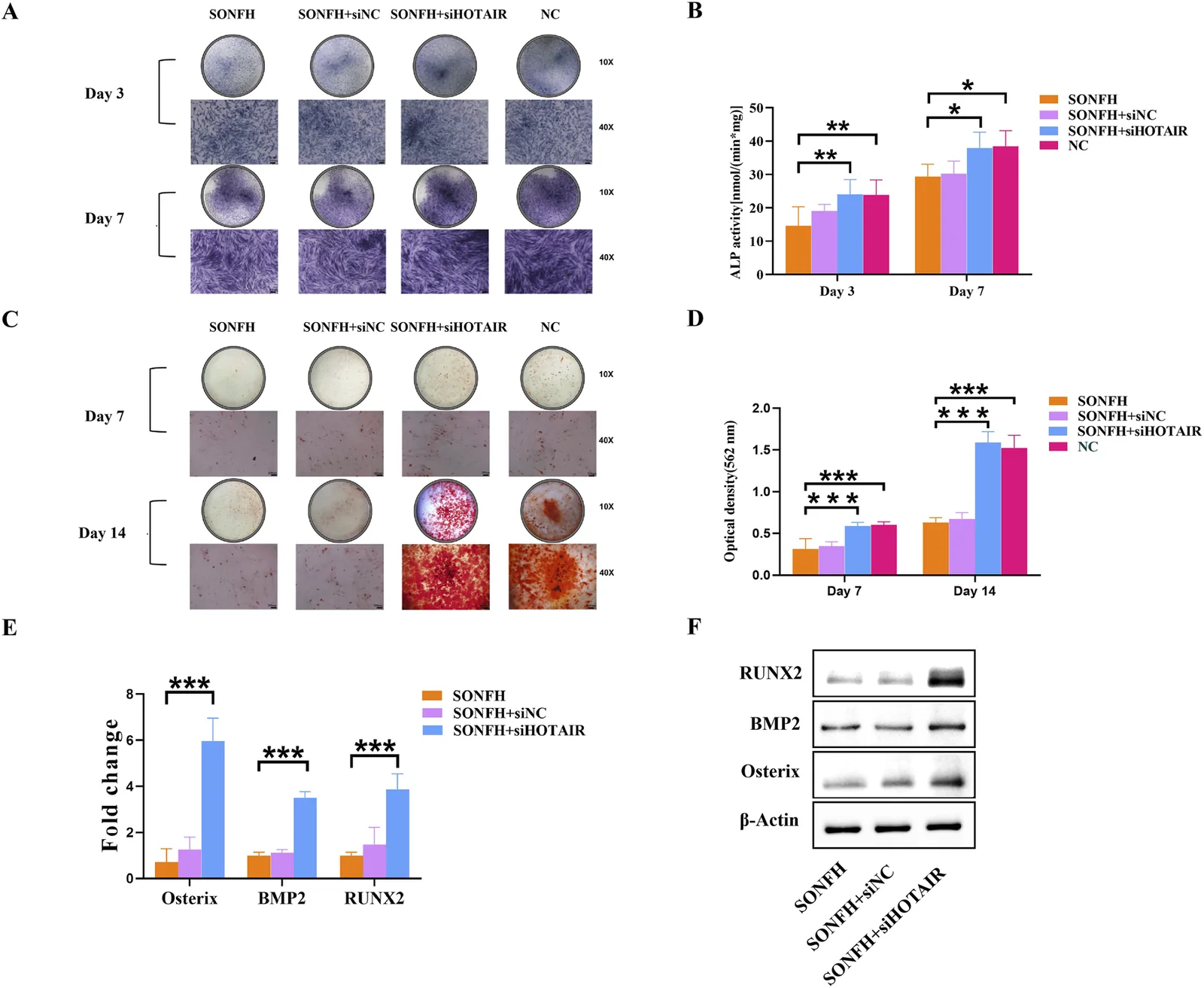
HOTAIR silencing promotes osteogenic differentiation of SONFH-BMSCs *in vitro*. **(A)** ALP staining of FNF-derived control BMSCs (NC), untreated SONFH-BMSCs, SONFH si-NC, and SONFH si-HOTAIR groups at days 3 and 7. **(B)** Quantitative analysis of ALP activity at days 3 and 7. **(C)** ARS staining at days 7 and 14. **(D)** Quantification of mineralization by CPC extraction followed by absorbance measurement at 562 nm. **(E)** qRT-PCR analysis of the expression of the osteogenesis-related genes, Osterix, BMP2, and RUNX2. **(F)** Western blot analysis of the osteogenic-related proteins, Osterix, BMP2, and RUNX2. Data are presented as mean ± SD from three independent biological replicates using BMSCs from independent donors, with three technical replicates per experiment. (*P < 0.05; **P < 0.01; ***P < 0.001).

Oil Red O staining on days 7 and 14 indicated that si-HOTAIR markedly inhibited intracellular lipid accumulation compared with untreated SONFH-BMSCs and si-NC controls (Figure 3A). Quantitative analysis confirmed that HOTAIR silencing significantly reduced lipid droplet formation at both day 7 (P < 0.01) and day 14 (P < 0.001; Figure 3B). qRT-PCR analysis further demonstrated that si-HOTAIR significantly downregulated the mRNA expression of the adipogenic markers PPARγ (P < 0.01), C/EBPα (P < 0.01), and Adipsin (P < 0.01) compared with untreated SONFH-BMSCs and si-NC controls (Figure 3C). Western blot results were consistent with qRT-PCR (Figure 3D). These findings indicated that HOTAIR silencing promoted osteogenic differentiation and inhibited adipogenic differentiation of BMSCs from patients with SONFH.

**FIGURE 3:**
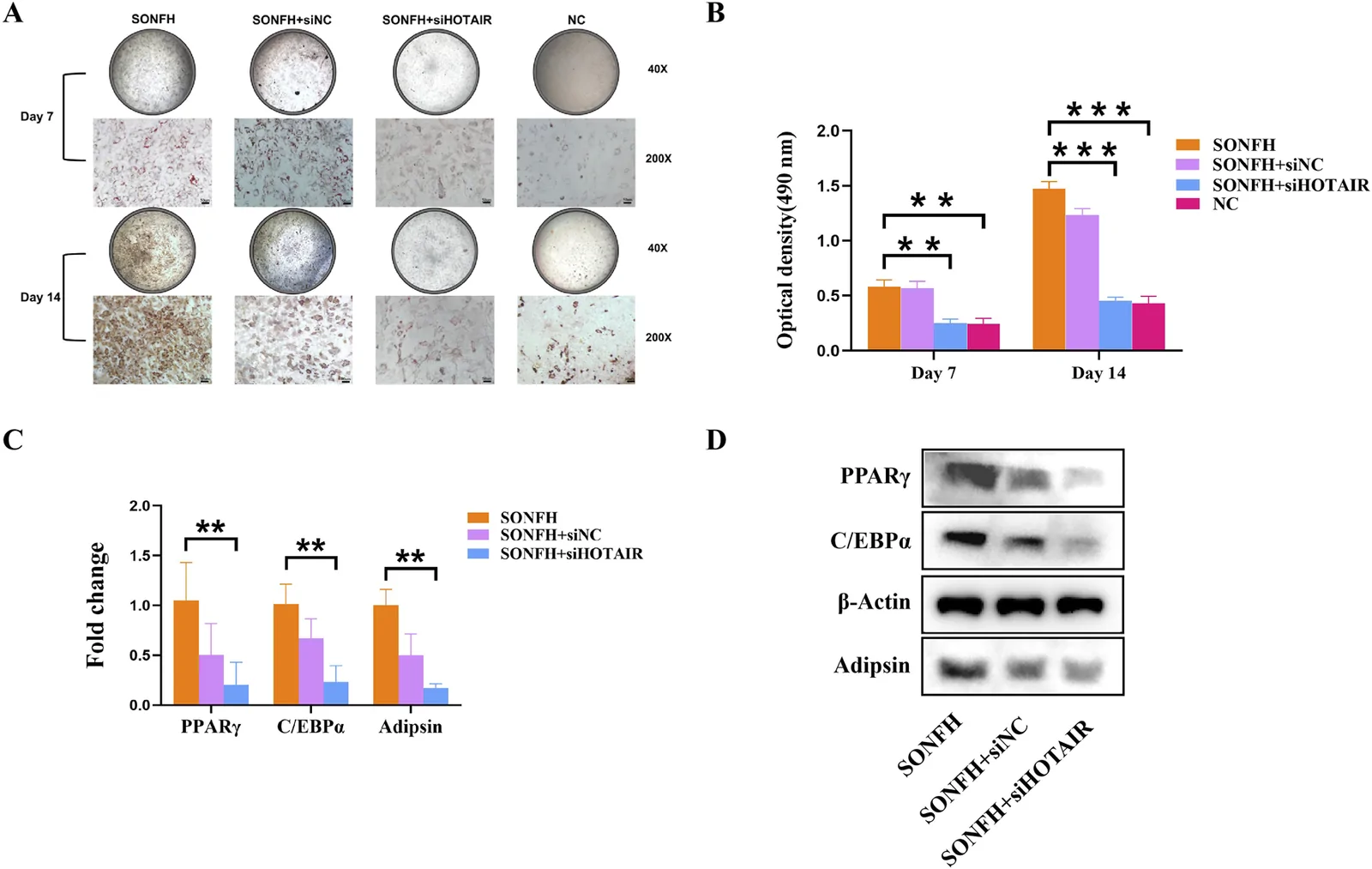
HOTAIR silencing inhibits adipogenic differentiation of SONFH-BMSCs *in vitro*. **(A)** Oil Red O staining of FNF-derived control BMSCs (NC), untreated SONFH-BMSCs, SONFH si-NC, and SONFH si-HOTAIR groups at days 7 and 14. **(B)** Quantitative analysis of Oil Red O staining at days 7 and 14. **(C)** qRT-PCR analysis of PPARγ, C/EBPα, and Adipsin mRNA levels. Expression was normalized to GAPDH and is shown compared with untreated SONFH-BMSCs and si-NC controls. **(D)** Western blot analysis of PPARγ, C/EBPα, and Adipsin. Data are presented as mean ± SD from three independent biological replicates using BMSCs from independent donors, with three technical replicates per experiment. (*P < 0.05; **P < 0.01; ***P < 0.001).

### miR-217 is predicted to bind both HOTAIR and DKK1

To elucidate the molecular mechanism by which HOTAIR regulates the differentiation imbalance in SONFH-BMSCs, we used a bioinformatic approach to identify candidate miRNAs that interact with HOTAIR. Five lncRNA-binding prediction databases—AnnoLnc (http://annolnc.cbi.pku.edu.cn/) (Ke et al., 2020), miRcode (http://www.mircode.org/) (Jeggari et al., 2012), StarBase (http://starbase.sysu.edu.cn/) (Zhou et al., 2026), miRDB (http://www.mirdb.org/) (Chen and Wang, 2020), and LncBase (http://carolina.imis.athena-innovation.gr/diana_tools/web/index.php) (Paraskevopoulou et al., 2016)—were initially screened. Among these, LncBase did not yield a usable prediction for HOTAIR and was excluded from further analysis. The remaining four databases collectively predicted multiple HOTAIR-interacting miRNAs. Notably, miR-217 was the only miRNA identified using AnnoLnc, miRcode, and StarBase simultaneously (Figure 4A). We therefore selected miR-217 for further investigation. The predicted binding site of miR-217 on HOTAIR is shown in Figure 4B. In parallel, TargetScan (McGeary et al., 2019) analysis identified a conserved miR-217 binding site within the 3′-UTR of DKK1 (Figure 4C), suggesting that HOTAIR and DKK1 may compete for miR-217 binding in a ceRNA regulatory network.

**FIGURE 4:**
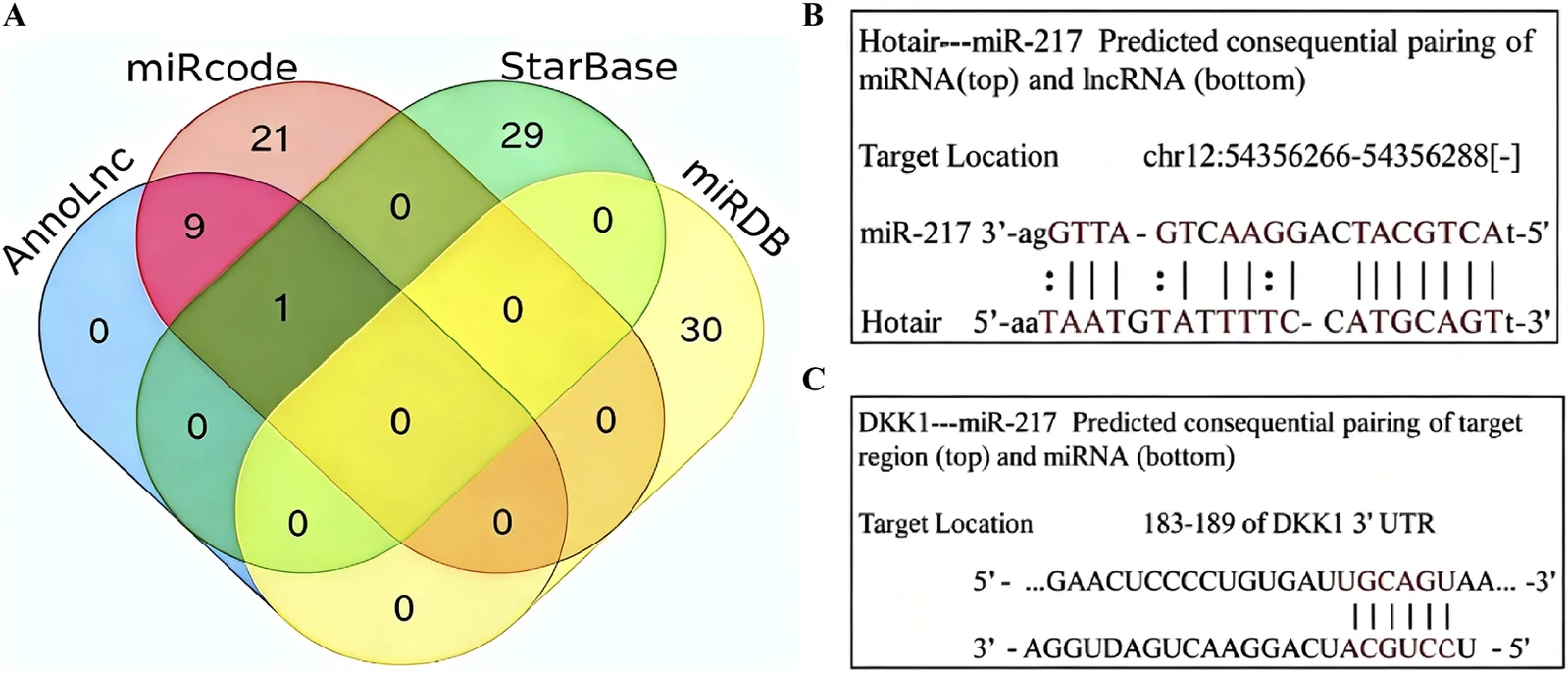
Identification of miR-217 as a candidate mediator interacting with HOTAIR and DKK1. **(A)** Venn diagram showing overlap of HOTAIR-targeting miRNAs predicted using AnnoLnc, miRcode, StarBase, and miRDB. **(B)** Predicted base-pairing between HOTAIR and miR-217. **(C)** Predicted base-pairing between the DKK1 3′-UTR and miR-217, as identified using TargetScan.

### miR-217 is downregulated in SONFH-BMSCs and directly targets HOTAIR and DKK1

qRT-PCR validation demonstrated that miR-217 was significantly downregulated in BMSCs from SONFH patients compared with controls (P < 0.01; Figure 5A). Moreover, Pearson correlation analysis identified a significant negative correlation between miR-217 expression and the transcript levels of both HOTAIR (Figure 5B) and DKK1 (Figure 5C) expression, consistent with a potential ceRNA regulatory relationship.

**FIGURE 5:**
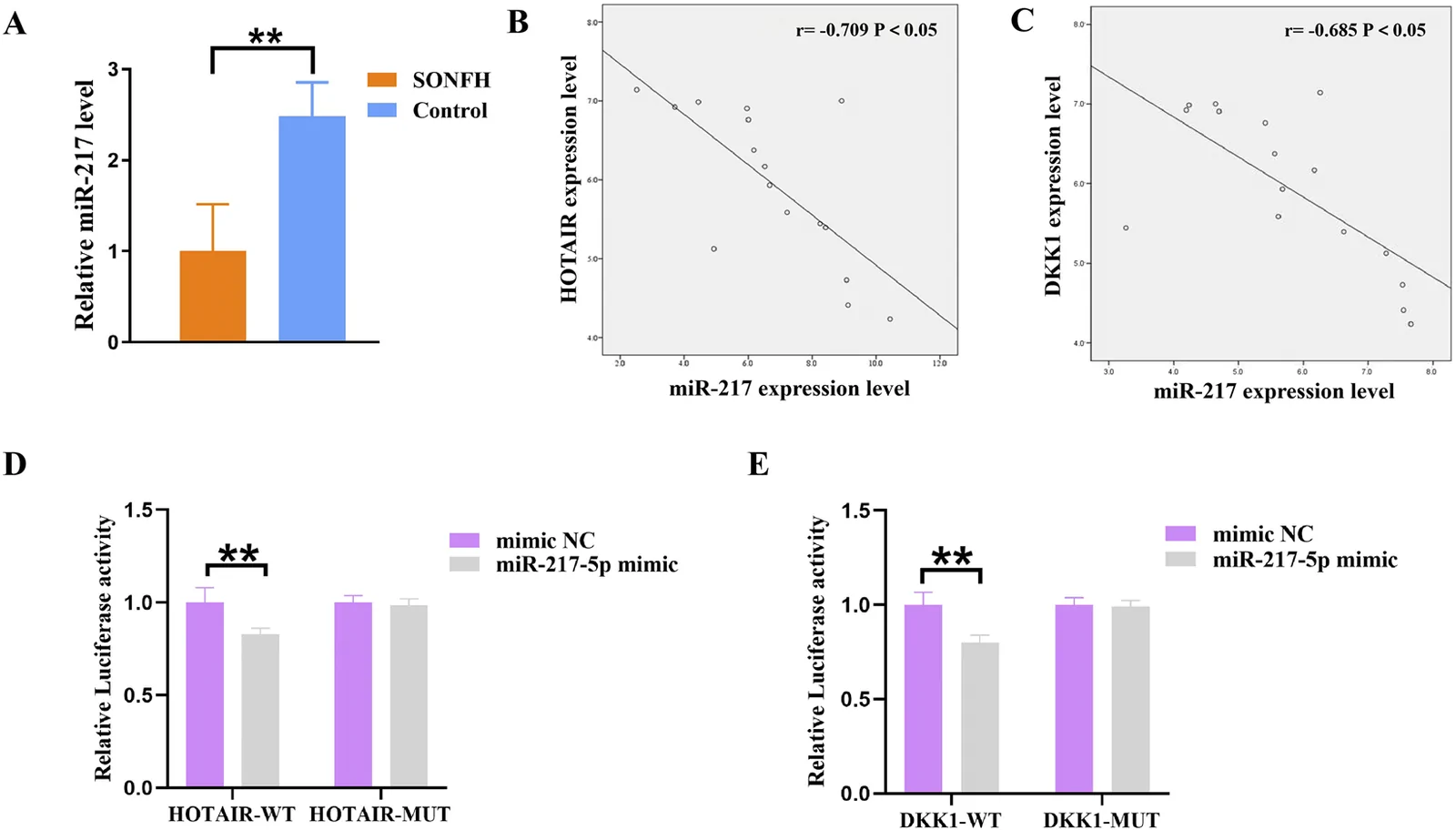
miR-217 is downregulated in SONFH-BMSCs and directly targets HOTAIR and DKK1. **(A)** qRT-PCR analysis of miR-217 expression in BMSCs from SONFH patients (n = 11) and controls (n = 10). Expression was normalized to U6. **(B)** Pearson correlation analysis between miR-217 and HOTAIR expression. **(C)** Pearson correlation analysis between miR-217 and DKK1 expression. **(D)** Dual-luciferase reporter assay validating the direct interaction between miR-217 and the predicted binding sequence of HOTAIR. HEK-293T cells were co-transfected with miR-217 mimics or mimic NC together with wild-type or mutant HOTAIR reporter constructs. **(E)** Dual-luciferase reporter assay for DKK1 3′-UTR-miR-217 interaction. Data are presented as mean ± SD from three independent transfection experiments (n = 3). (*P < 0.05; **P < 0.01).

To determine whether miR-217 directly targets HOTAIR and DKK1, dual-luciferase reporter assays were performed. Wild-type (HOTAIR-WT and DKK1-WT) and mutant (HOTAIR-MUT and DKK1-MUT) luciferase reporter vectors were co-transfected into HEK-293T cells with miR-217 mimics or mimic NC. Co-transfection of miR-217 mimics with HOTAIR-WT significantly reduced relative luciferase activity compared with the mimic NC control (P < 0.01), whereas mutation of the predicted binding site (HOTAIR-MUT) abolished this effect (P > 0.05; Figure 5D). Similarly, miR-217 mimics significantly suppressed the luciferase activity of DKK1-WT (P < 0.01) but had no significant effect on DKK1-MUT (P > 0.05; Figure 5E). These findings demonstrate that miR-217 directly interacts with HOTAIR and DKK1 transcripts, supporting the involvement of a HOTAIR/miR-217/DKK1 regulatory axis.

### HOTAIR regulates DKK1 expression via sponging miR-217

To determine whether HOTAIR regulates DKK1 through miR-217 sponging, we first examined the effect of HOTAIR modulation on miR-217 expression. Silencing HOTAIR with si-HOTAIR significantly increased miR-217 levels (P < 0.01; Figure 6A). We next performed rescue experiments to assess whether miR-217 mediates the effect of HOTAIR on DKK1. Knockdown of HOTAIR significantly reduced DKK1 mRNA and protein expression; notably, co-transfection with a miR-217 inhibitor partially but significantly reversed this reduction, restoring DKK1 expression toward control levels (Figures 6B,C).

**FIGURE 6:**
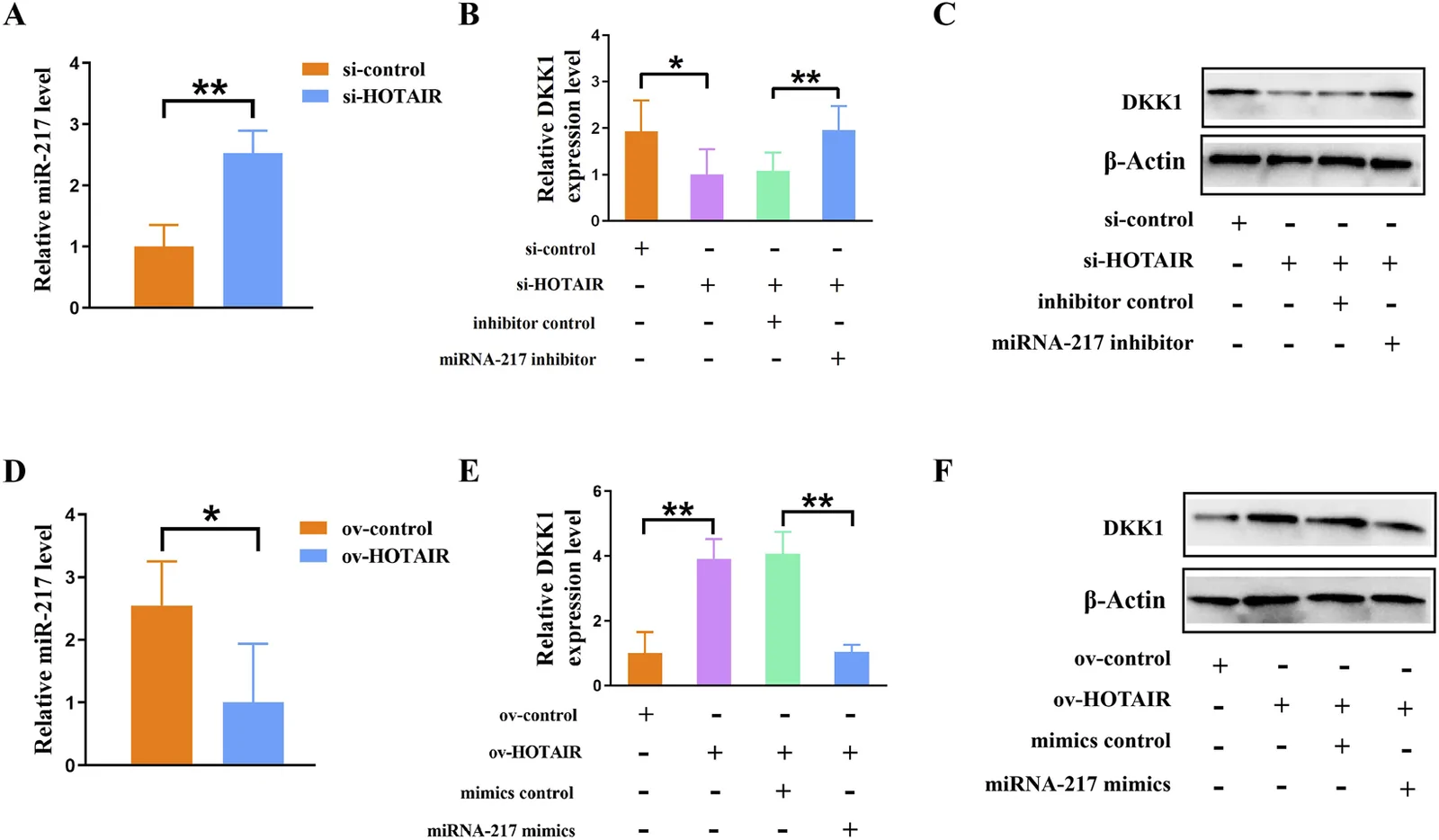
HOTAIR regulates DKK1 expression by sponging miR-217 in SONFH-BMSCs. **(A)** qRT-PCR analysis of miR-217 expression following HOTAIR knockdown with si-HOTAIR. **(B)** qRT-PCR analysis of DKK1 mRNA levels following HOTAIR knockdown with or without miR-217 inhibitor co-transfection. **(C)** Western blot images of DKK1 protein expression under the same conditions. **(D)** qRT-PCR analysis of miR-217 expression following HOTAIR overexpression with ov-HOTAIR. **(E)** qRT-PCR analysis of DKK1 mRNA levels following HOTAIR overexpression with or without miR-217 mimic co-transfection. **(F)** Western blot images of DKK1 protein expression under the same conditions. Data are presented as mean ± SD from three independent biological replicates. (*P < 0.05; **P < 0.01).

Conversely, overexpression of HOTAIR (ov-HOTAIR) significantly decreased miR-217 levels (P < 0.05; Figure 6D), confirming that HOTAIR negatively regulates miR-217 availability. HOTAIR overexpression significantly increased DKK1 mRNA and protein levels (P < 0.05), and this upregulation was partially attenuated by co-transfection with miR-217 mimics (P < 0.01; Figures 6E,F).

Collectively, these results support a model in which HOTAIR functions as a ceRNA that positively regulates DKK1 primarily, although not exclusively, through competitive sequestration of miR-217.

### HOTAIR silencing activates Wnt/β-catenin signaling via the miR-217/DKK1 axis

DKK1 is a well-established antagonist of the canonical Wnt/β-catenin pathway. We therefore examined whether HOTAIR-mediated regulation of DKK1 through miR-217 affects downstream Wnt/β-catenin signaling. OCT4 was evaluated only as an associated differentiation-status marker rather than as a direct mechanistic mediator. qRT-PCR and Western blot analyses revealed that HOTAIR silencing was associated with decreased OCT4 levels while concurrently increasing β-catenin expression (P < 0.05; Figures 7A,B). Conversely, HOTAIR overexpression significantly increased OCT4 levels and decreased β-catenin expression (P < 0.01; Figures 7C,D). These reciprocal changes are consistent with the ceRNA model: HOTAIR-mediated upregulation of DKK1 via miR-217 sponging inhibits Wnt/β-catenin signaling, whereas HOTAIR silencing relieves this inhibition, leading to increased β-catenin expression and enhanced Wnt/β-catenin-associated signaling.

**FIGURE 7:**
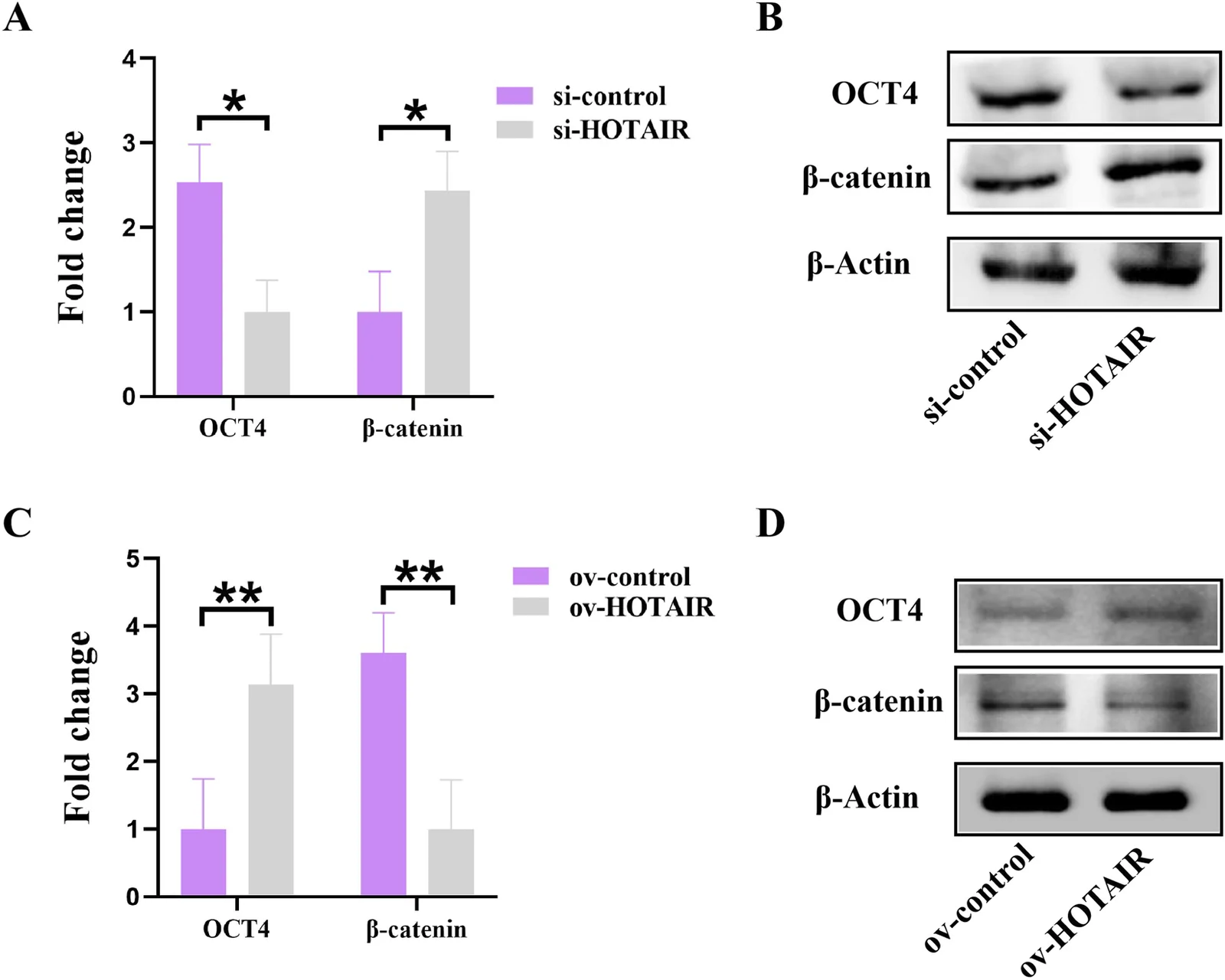
HOTAIR silencing modulates Wnt/β-catenin signaling through the miR-217/DKK1 axis. **(A)** qRT-PCR analysis of OCT4 and β-catenin mRNA expression in BMSCs transfected with si-HOTAIR or si-control. **(B)** Western blot analysis of OCT4 and β-catenin protein levels in si-HOTAIR and si-control-transfected BMSCs. **(C)** qRT-PCR analysis of OCT4 and β-catenin mRNA expression in BMSCs transfected with ov-HOTAIR or ov-control. **(D)** Western blot analysis and quantification of OCT4 and β-catenin protein levels in ov-HOTAIR- and ov-control-transfected BMSCs. Data are presented as mean ± SD from three independent biological replicates. (*P < 0.05; **P < 0.01; ***P < 0.001).

## Discussion

SONFH is fundamentally characterized by a profound disruption of bone homeostasis, in which BMSCs exhibit impaired osteogenic differentiation and aberrantly enhanced adipogenic commitment (Xiang et al., 2025). This imbalance is widely recognized as a central pathological driver of trabecular bone loss, marrow fat accumulation, and progressive femoral head collapse. In the present study, we used BMSCs derived from patients with FNF as controls as these cells provide a clinically relevant comparator representing non-glucocorticoid-exposed BMSCs from the same anatomical site and surgical setting. However, the molecular mechanisms linking epigenetic regulation, post-transcriptional control, and stem cell fate decisions in SONFH remain incompletely understood (Ma et al., 2026).

Accumulating evidence indicates that lncRNAs play critical roles in regulating MSC differentiation and skeletal homeostasis. Several lncRNAs, including DGCR5 (Jiang et al., 2023), FAR591 (Zhang et al., 2023), and MALAT1 (Huang et al., 2020), have been implicated in SONFH pathogenesis, highlighting the importance of noncoding RNA networks in this disease. In particular, lncRNAs can act as ceRNAs to sponge miRNAs and modulate downstream gene expression, forming ceRNA networks that contribute to the progression of SONFH and other osteonecrosis-related disorders (Sun et al., 2025).

HOTAIR has been implicated in inhibiting osteogenic differentiation in skeletal disorders, including osteoporosis (Shen et al., 2019). Our prior work identified aberrantly elevated HOTAIR expression specifically in SONFH patient-derived BMSCs (Wang et al., 2018). The present study extends these findings by demonstrating that HOTAIR silencing can simultaneously restore osteogenic differentiation and suppress excessive adipogenesis in patient-derived BMSCs *in vitro*. These results establish HOTAIR as a functionally relevant regulator in SONFH-associated differentiation imbalance.

Mechanistically, our bioinformatic analysis and functional assays delineate a ceRNA regulatory axis in which HOTAIR competitively binds miR-217, thereby upregulating DKK1, a key secreted antagonist of the canonical Wnt/β-catenin pathway. We confirmed that miR-217 levels are inversely correlated with both HOTAIR and DKK1 expression across individual patient samples, and dual-luciferase reporter assays validated both HOTAIR and DKK1 as direct targets of miR-217. Rescue experiments further demonstrated that HOTAIR regulates DKK1 primarily through miR-217 sponging, although the partial nature of the rescue suggests that additional miRNAs or regulatory mechanisms may also contribute downstream of HOTAIR. This ceRNA-mediated regulation converges on the Wnt/β-catenin pathway: HOTAIR silencing increases miR-217 availability, reduces DKK1 expression, and consequently activates β-catenin signaling, thereby favoring osteogenic differentiation. The observed changes in OCT4 expression may reflect altered differentiation status rather than a direct downstream mediator of this regulatory axis. Although individual molecular interactions within this axis have been described in other contexts, the present study provides integrated evidence supporting the involvement of this ceRNA network in SONFH-associated osteogenic/adipogenic differentiation imbalance *in vitro*.

Several limitations of this study should be acknowledged. First, our findings are based entirely on *in vitro* experiments and do not recapitulate the complex *in vivo* microenvironment of SONFH, including local hypoxia, chronic inflammation, and vascular impairment. Therefore, the functional rescue observed in patient-derived BMSCs should be viewed as a proof-of-concept requiring rigorous validation in preclinical animal models. Moreover, although HOTAIR inhibition demonstrated the potential to restore osteogenic–adipogenic balance in SONFH-BMSCs, this study did not evaluate the therapeutic efficacy of HOTAIR-targeted intervention *in vivo* or in clinical settings. Future studies incorporating appropriate animal models and intervention strategies are required to determine whether modulation of the HOTAIR/miR-217/DKK1 axis can provide therapeutic benefits for SONFH. Second, the relatively modest sample size (n = 11 SONFH; n = 10 controls) may limit the generalizability of our correlative analyses, warranting validation in larger multi-center cohorts. Third, although dual-luciferase reporter and rescue experiments supported the HOTAIR/miR-217/DKK1 regulatory axis, endogenous interaction assays such as AGO2-RIP were not performed. Therefore, further studies are required to characterize the physiological assembly of this ceRNA network. The incomplete reversion of DKK1 levels in our rescue experiments also suggests that additional HOTAIR-interacting miRNAs or non-ceRNA mechanisms may contribute.

## Conclusion

In conclusion, our findings suggest that HOTAIR is an important regulator of osteogenic–adipogenic imbalance in SONFH-BMSCs through the miR-217/DKK1/Wnt/β-catenin axis. These findings provide new mechanistic insights into SONFH pathogenesis and highlight the importance of ceRNA-mediated regulation in BMSC fate determination.

## Data Availability

All data generated or analyzed during this study are included in this published article and its supplementary information files. Raw datasets are available from the corresponding author upon reasonable request.

## Supplementary material

SUPPLEMENTARY FIGURE S1 Validation of HOTAIR knockdown efficiency in SONFH-BMSCs. Relative HOTAIR mRNA levels were detected by qRT-PCR in SONFH-BMSCs or transfected with si-NC or si-HOTAIR. GAPDH was used as the internal reference. Data are presented as mean ± SD from three independent biological replicates. **P < 0.01.
